# Pressure-induced nano-crystallization of silicate garnets from glass

**DOI:** 10.1038/ncomms13753

**Published:** 2016-12-07

**Authors:** T. Irifune, K. Kawakami, T. Arimoto, H. Ohfuji, T. Kunimoto, T. Shinmei

**Affiliations:** 1 Geodynamics Research Center, Ehime University, Matsuyama 790–8577, Japan; 2 Earth Life Science Institute, Tokyo Institute of Technology, Tokyo 152–8550, Japan

## Abstract

Transparent ceramics are important for scientific and industrial applications because of the superior optical and mechanical properties. It has been suggested that optical transparency and mechanical strength are substantially enhanced if transparent ceramics with nano-crystals are available. However, synthesis of the highly transparent nano-crystalline ceramics has been difficult using conventional sintering techniques at relatively low pressures. Here we show direct conversion from bulk glass starting material in mutianvil high-pressure apparatus leads to pore-free nano-polycrystalline silicate garnet at pressures above ∼10 GPa in a limited temperature range around 1,400 °C. The synthesized nano-polycrystalline garnet is optically as transparent as the single crystal for almost the entire visible light range and harder than the single crystal by ∼30%. The ultrahigh-pressure conversion technique should provide novel functional ceramics having various crystal structures, including those of high-pressure phases, as well as ideal specimens for some mineral physics applications.

## Abstract (web summary)

It is suggested that the optical and mechanical properties of transparent ceramics become very favourable if they can be synthesized as nanocrystals. Here, the authors report direct conversion of bulk glass starting material to pore-free nano-polycrystalline silicate garnet at high pressure and temperature.

Conventional ceramics are made of inorganic crystals, such as oxides and silicates, produced by firing raw materials in an oven, which are generally hard but fragile partly due to the presence of a large fraction of inter-crystalline pores. The presence of the pores, in addition to ionic and covalent bonding nature of these crystals, makes the ceramics good electrical and thermal insulators, which have been utilized for potteries, tiles, bricks and so on from ancient time. These ceramics are opaque even if the crystals themselves are transparent because of scattering of lights by the pores or additive materials.

Transparent ceramics have been synthesized by various methods, such as hot isostatic pressing, Spark Plasma Sintering and crystallization from glass at ambient pressure or relatively low pressures far below 10 GPa, by minimizing the light-scattering sources^1^. Highly transparent ceramics having transparencies close to those of the corresponding single crystals, mostly with cubic crystallographic symmetry, have been synthesized and commercialized as optical lenses, laser amplifiers and so on^1^. These transparent ceramics are made of crystals with grain sizes greater than a few hundred nanometres mainly due to grain growth at high temperature, while synthesis of such transparent ceramics with purely nano-crystals, having grain sizes <100 nm (ref. 2), at lower temperatures have not been realized^3^,^4^,^5^,^6^,^7^,^8^ mainly because of the difficulty in removing inter-grain residual pores. However, it is predicted that the optical transparency will be significantly improved for the ceramics even with non-cubic crystals, if those made of nano-crystals are available^9^,^10^. Moreover, the hardness of such ceramics is suggested to be also enhanced in the nano-crystalline regime^7^,^11^.

We succeeded to synthesize ultrahard nano-polycrystalline diamond (NPD) by direct conversion from graphite at high pressure and high temperature using multianvil apparatus^12^. Relatively large NPD rod samples up to ∼1 cm in both diameter and length can now be produced using a large-volume multianvil apparatus^13^, and have been successfully applied for various industrial tools as the first commercialized products obtained in the ultrahigh pressure regime (generally defined as the pressure range of above 10 GPa in materials synthesis). By applying a similar technique, we recently reported synthesis of nano-polycrystalline stishovite^14^ (a high-pressure polymorph of SiO_2_ quartz) from silica glass with both high hardness and toughness at a pressure of 15 GPa and temperatures above 1,000 °C. However, thus-synthesized samples exhibited only poor optical transparency.

Here we report the results of a systematic investigation of synthesis and characterization of sintered bodies of polycrystalline Ca_3_Al_2_Si_3_O_12_ grossular garnet at pressures to 15 GPa and temperatures to 1,600 °C under perfectly dry conditions and quasi-hydrostatic pressures in multianvil high-pressure apparatus. Nano-polycrystalline grossular, which is as transparent as the single crystal grossular and harder than the single crystal, is obtained at pressures above ∼10 GPa at the modest temperatures around 1,400 °C. Similarly, transparent garnets with various chemical compositions are also synthesized under these pressure and temperature conditions. The successful synthesis of the super-transparent nano-polycrystalline garnet is attributed to rapid nucleation of the crystals followed by their slow grain growth under such ultrahigh pressures at the modest temperatures.

## Results

### Microstructure and grain size of synthesized grossular

**Figure 1:**
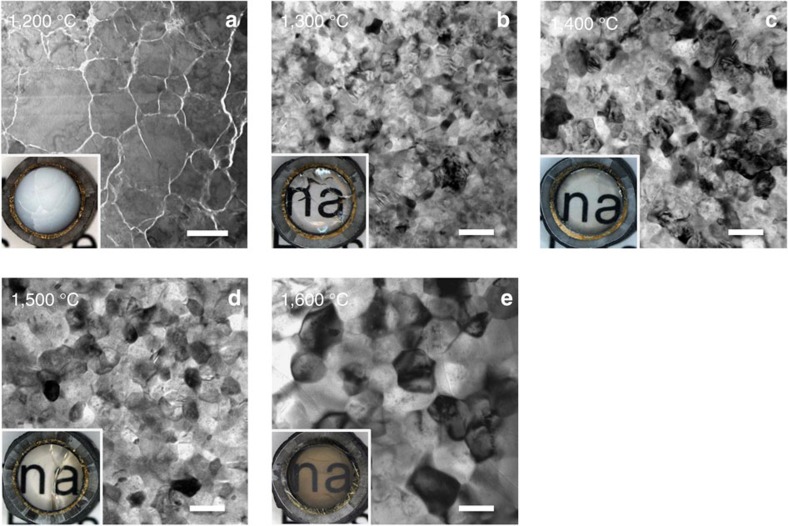
TEM and optical microscope images of polycrystalline grossular samples synthesized at 15 GPa. (**a**) Grossular with the large grains up to a few micrometres with many inter-grain crack openings formed at 1,200 °C. (**b**–**d**) Nano-polycrystalline grossular with high optical transparency formed at temperatures 1,300–1,500 °C. (**e**) Grossular synthesized at 1,600 °C was slightly dark and brown in colour with some grain growth to ∼200 nm. The grossular bulk samples have diameters of ∼4 mm after the recovery, as shown by the inlet optical microscope images, where the cross-sections of surrounding metal capsule and MgO sleeve are also seen. The scale bar in **a** is 2 μm, while those in other pictures are 200 nm.

**Figure 2:**
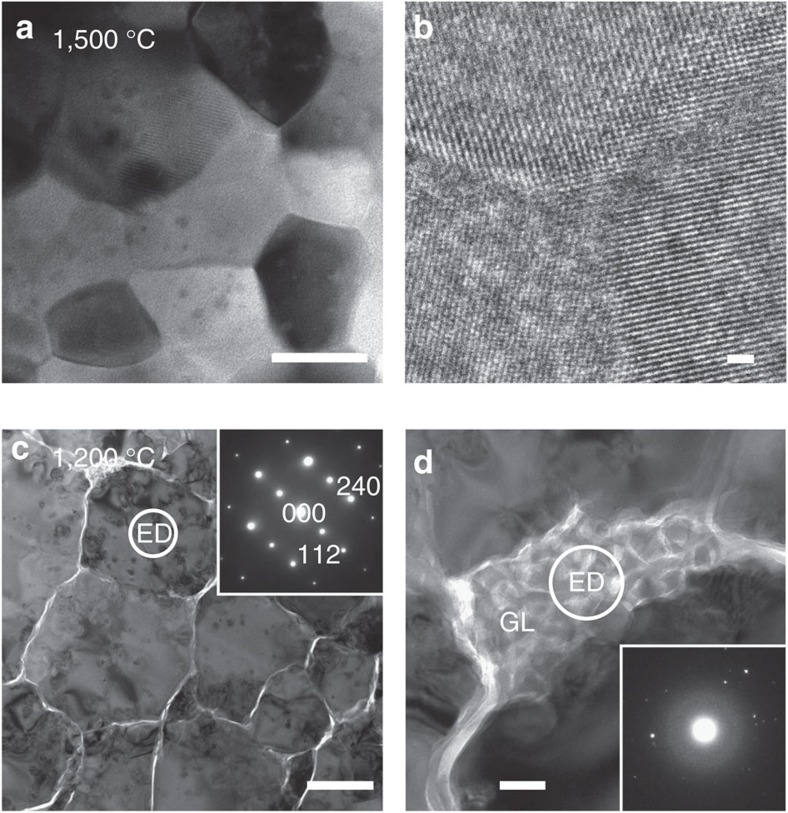
Detailed TEM images of polycrystalline grossular samples synthesized at 1,500 °C and 1,200 °C at 15 GPa. The sample synthesized at 1,500 °C consists of nano-crystalline grains that are tightly contacted with each other with virtually no pores or openings (**a**); scale bar, 100 nm. High-resolution TEM image of a triple junction indicates that the individual grains are crystalline up to grain boundary and closely interlocked (**b**); scale bar, 1 nm. The sample synthesized at 1,200 °C consists of microcrystalline grains with a number of tangled dislocations and inter-grain micro crack openings (**c**); scale bar, 1 μm. A mixture of glass with the grossular composition and nano-crystalline grossular embryos was found at grain boundaries (**d**); scale bar, 100 nm. The circles labelled with ED (electron diffraction) indicate the selected areas (by apertures) from which ED images were taken (shown as an inlet of **c**,**d**). The latter ED taken from the region labelled GL shows a diffuse halo characteristic of glass (amorphous) material, in addition to diffraction spots from the coexisting nano-crystals.

Transmission electron microscope (TEM) and optical microscope images of the samples synthesized at 15 GPa and at temperatures of 1,200–1,600 °C are shown in Fig. 1. The sample recovered from the run at 1,100 °C was colourless and transparent but remained glass. On increasing temperature, the recovered sample got devitrified to milky white at 1,200 °C (Fig. 1a, inlet), and again became colourless and highly transparent at temperatures of 1,300, 1,400 and 1,500 °C (Fig. 1b–d inlet). The sample synthesized at 1,600 °C was also transparent but slightly dark and brown in colour (Fig. 1e, inlet). All of these samples synthesized at temperatures 1,200–1,600 °C were identified as pure grossular by X-ray diffraction measurements, and were found free of residual pores by TEM observations (Fig. 2a,b) and by density measurements using the Archimedes method (Methods).

TEM image shows the devitrified grossular sample obtained at 1,200 °C is composed of relatively large grains of grossular up to a few micrometres, having many dislocations and inter-grain crack openings (Fig. 2c). A small amount of remnant glass was also observed along the grain boundaries (Fig. 2d). The large grain size at such a relatively low temperature is attributed to the slow nucleation of garnet in homogeneous glass just above the glass transition temperature^15^ of around 1,150 °C at this pressure, followed by crystal growth. Nano-polycrystalline grossular with average grain sizes <100 nm was found to form at higher temperatures of 1,300–1,500 °C at 15 GPa (Fig. 1b–d and Supplementary Table 1). This is probably due to the fast nucleation above some threshold temperature between 1,200 and 1,300 °C, which resulted in the instantaneous formation of a large number of garnet crystallites, followed by slow grain growth because of the dry and ultrahigh pressure conditions. In contrast, significant grain growth of up to ∼200 nm was noted for the sample synthesized at a further high temperature of 1,600 °C (Fig. 1e).

Similar temperature dependency in grain size of grossular was observed at 10 GPa (and at 12 GPa); nano-polycrystalline garnets formed only at the modest temperatures of 1,400 °C, while those synthesized at 1,200–1,300 and 1,500–1,600 °C had larger grains of 150–200 nm. In contrast, the average grain sizes of grossular synthesized at 8 GPa and at 1,200–1,400 °C were substantially larger than 100 nm, and those synthesized at 5 GPa were even much larger; 33±13 μm at 1,200 °C; 5±2 μm at 1,400 °C; and 7±3 μm at 1,600 °C (Supplementary Table 1). The latter results at the lowest pressure may be due to the smaller nucleation rates near the phase boundary between grossular and the low pressure phases, combined with the faster atomic diffusions at such a relatively low pressure, causing substantial crystal growth in the glass followed by grain growth.

**Figure 3:**
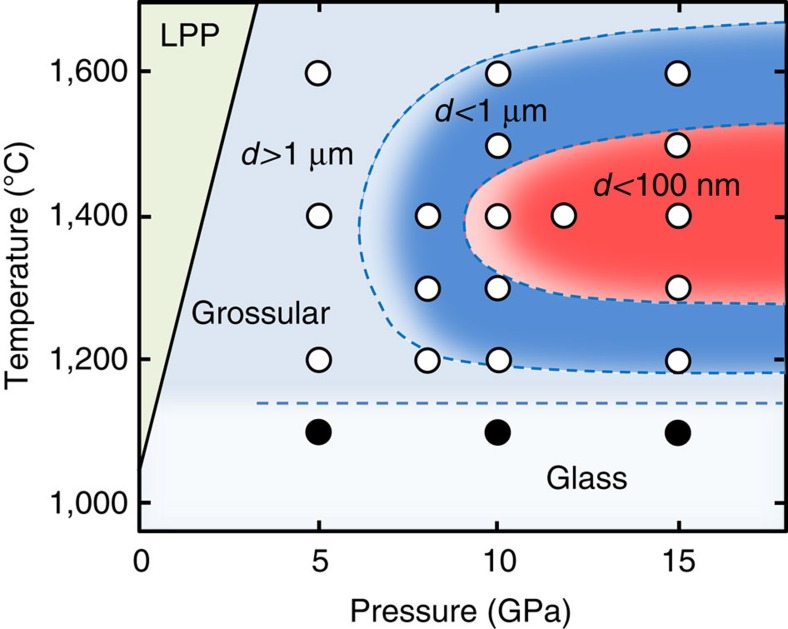
Pressure and temperature conditions for the synthesis of grossular. Polycrystalline grossular with grain sizes <1 μm is shown in the blue region. Highly transparent nano-polycrystalline garnet was obtained only in the red region at around 1,400 °C and at pressures at and above 10 GPa, while the samples remained glass at 1,100 °C. Note that these results on the grain size may change with the heating time, and the dotted boundaries are applicable to the runs with 2 h heating at the given pressures and temperatures. LPP, low-pressure phases (anorthite, gehlenite and wollastonite) of grossular^38^.

Figure 3 summarizes the pressure and temperature conditions, where grossular crystallizes in various grain sizes from the glass starting material. To obtain nano-polycrystalline garnet, a fast nucleation of garnet without subsequent grain growth is required, which can only be achieved under limited conditions of pressures at and above ∼10 GPa and temperatures around 1,400 °C for grossular. Excluding absorbed water during the ultrahigh-pressure synthesis is also important, as the presence of even a trace amount of water can yield significantly faster atomic diffusions^16^, causing rapid grain growth. The presence of water may also result in poor grain boundary adhesion and the formation of inter-grain defective regions, which should be avoided in making transparent ceramics. For this purpose, using bulk glass sample, rather than powdered glass, is essentially important to minimize the surface area of the starting sample. Indeed, our attempts to make nano-polycrystalline garnet using powdered glass starting materials failed, and the garnets synthesized under similar pressure and temperature conditions were made of crystals with average grain sizes always >1 μm, in spite of careful treatments of the sample and furnace assembly to exclude absorbed water^17^.

### Hardness versus grain size

**Figure 4:**
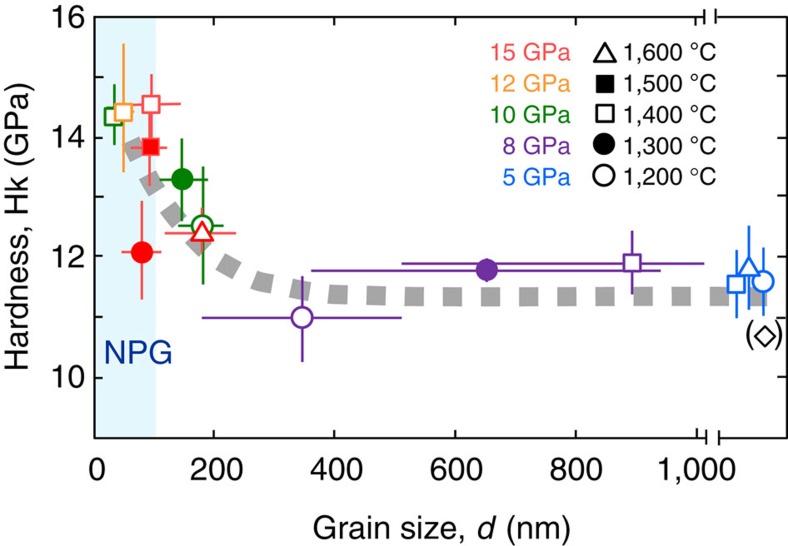
Knoop hardness versus grain size of the polycrystalline grossular. The Knoop hardness (Hk) increases with reducing grain size (*d*) particularly those below 200 nm, suggesting the Hall–Petch effect is applicable to nano-polycrystalline grossular, while no obvious relations of the hardness with either pressure or temperature are noted. Pressures are shown in different colours, while temperatures are shown by different symbols, as indicated in the figure. Open black diamond symbol in a parenthesis represents the hardness of a single crystal of natural grossular with a small (<1 wt%) amount of FeO and MnO (Methods). The hardness data of the samples with *d*>1,000 nm do not correspond to the scale of the horizontal axis, but are plotted in the order of the grain size. The shaded area indicated by NPG (nano-polycrystalline garnet) corresponds to the nano-crystalline region. The measured values of hardness and grain size are listed in Supplementary Table 1, with s.d.'s shown by error bars in this figure.

Figure 4 shows Knoop hardness (Hk) of the polycrystalline grossular samples synthesized in the present study as a function of grain size (*d*). It is clearly seen that the hardness increases by ∼30% with reducing grain size, from Hk=11.3±0.5 GPa with *d*>200 nm to ∼14.5 GPa at *d*=30–50 nm, showing that the Hall–Petch effect^18^,^19^,^20^ known for metals is also applied to some ceramics in the nano-crystalline regime. This is consistent with the recent results on polycrystalline MgAl_2_O_4_ spinel^11^ that concluded the Hall–Petch effect is valid down to the grain size of 30 nm, although there is controversy on the applicable grain size for this effect in ceramics^21^. The latter result on MgO periclase may be due to insufficient sintering of nano-crystals, and our results on pore-free samples clearly demonstrate the Hall–Petch effect is applied to the nano-ceramics.

### Optical transmittance

**Figure 5:**
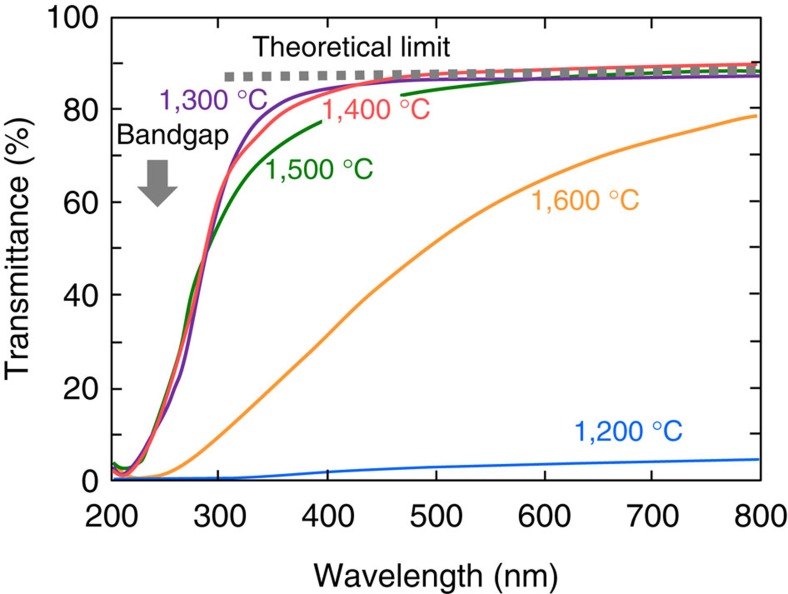
Transparency of polycrystalline grossular synthesized at 15 GPa. Transmittance of light through the polycrystalline grossular samples with a thickness of 1.0 mm, synthesized at 15 GPa and at 1,200–1,600 °C (Fig. 1). Dotted line shows the theoretical limit (86.7%) of the transmittance calculated from the refractive index of single-crystal grossular. The predicted bandgap (5.22 eV=238 nm in wavelength) of grossular is indicated by the arrow.

Figure 5 shows the transmittance of ultraviolet to visible light through the polycrystalline grossular samples with a thickness of 1.0 mm, synthesized at 15 GPa and at different temperatures. The theoretical limit (86.7%) of the transmittance of visible light predicted from the refractive index of single-crystal grossular (*n*=1.734; ref. 22) is also shown as a reference. The transmittances of the transparent samples synthesized at 1,300, 1,400 and 1,500 °C are very close to that of the theoretical limit in the visible light range between about 400 and 800 nm in wavelength. The gradual decrease of the transmittance for wavelengths shorter than ∼400 nm and the sharp decrease below ∼300 nm would be attributed to a combination of the scattering caused by trace amounts of inter-grain defective regions with similar dimensions to the grain sizes (30–50 nm) presumably formed on the release of pressure and the absorption due to the electronic bandgap of grossular (5.22 eV=238 nm; ref. 23). In contrast, the optical transmittance of the dark and brown transparent sample synthesized at 1,600 °C is substantially lower than the theoretical limit in this wavelength range, which is probably caused by the inter-grain defective regions equivalent to the grain sizes (around 100–200 nm) formed on the pressure release, which should significantly scatter the lights in the visible light range^9^,^10^. On the other hand, the very low transparency of the sample crystallized at the lowest temperature (1,200 °C) at 15 GPa is attributed to the scattering of light by a small amount of remnant glass and the openings along the grain boundaries due to incomplete sintering (Fig. 2c,d).

## Discussion

The present direct conversion method at ultrahigh pressure and high temperature using the bulk glass sample first succeeded in producing pore-free nano-crystalline ceramics with the optical transparency comparable to that of the corresponding single crystal. Although highly transparent ceramics have been developed^24^ and successfully applied to some lasers and optical devices^25^, those with single-phase crystals in the nano-crystalline regime have never been produced by either sintering techniques^3^,^4^,^5^,^6^,^7^,^24^ or crystallization from glass or amorphous phases^8^,^26^,^27^ under the ambient pressure and at relatively low pressures. On the other hand, the transparent nano-ceramics produced by these techniques have substantially low transparency relative to the single crystals in the visible light region^11^,^27^ mainly due to the presence of a number of residual pores and/or defective regions along grain boundaries. To remove these scattering sources, higher-temperature or longer-duration heating is required, which inevitably leads to grain growth at the low pressures and fails to produce nano-crystalline ceramics. In contrast, our ultrahigh-pressure conversion technique helps strengthening of inter-grain adhesion and also suppresses grain growth due to slower atomic diffusions even at high temperatures, leading to the ceramics with both super-transparency and nano-crystalline nature that have never been achieved in earlier studies.

**Figure 6:**
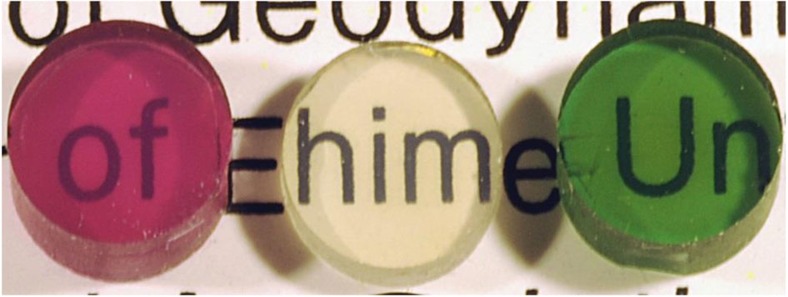
Transparent nano-polycrystalline garnets with various chemical compositions. Some examples of high-quality polycrystalline garnet synthesized at 15 GPa and at 1,400 °C, with a diameter of ∼4 mm and thickness of ∼2 mm; grossular with 2 mol% Ca_3_Cr_2_Si_3_O_12_ uvarovite (green), pure grossular (colourless) and Mg_3_Al_2_Si_3_O_12_ pyrope with 5 mol% knorringite Mg_3_Cr_2_Si_3_O_12_ (purple).

We confirmed the present ultrahigh-pressure conversion technique can be applied to other silicate garnets with different chemical compositions in producing similarly transparent polycrystalline samples (Fig. 6), which suggests highly transparent nano-crystalline ceramics may be produced wherever bulk glass starting materials are available. As the transparency of sintered polycrystalline materials is predicted to increase in the nano-crystalline regime even for those with optically anisotropic symmetry^9^,^10^, the present technique using ultrahigh pressure and bulk glass starting material under completely dry conditions should open the door to making novel transparent nano-crystalline ceramics with superior mechanical strength. Moreover, unlike conventional sintering techniques, the present method does not require the preparation of nano-crystalline powders, which should result in the novel transparent ceramics made of materials with a wide range of chemical compositions.

The present method also has great advantage in making transparent ceramics with high-pressure phases, which generally have higher values in density, elastic stiffness, hardness, refractive index and so on relative to those of the lower pressure phases but have been available mostly as powders or poorly sintered bodies with grain sizes far larger than 100 nm. Sintering of such high-pressure phases is difficult under the ambient pressure or at pressures lower than their stability limits, as they transform back to low-pressure phases or amorphous phases. Thus, transparent ceramics of high-pressure phases cannot in principle be produced using conventional methods at relatively low pressures. The present ultrahigh-pressure conversion technique is not only useful in making super-transparent ceramics with materials formed at the ambient pressure but also expands such ceramics to high-pressure phases with more densely packed crystal structures. In fact, all of the garnet specimens in our study are unstable at the ambient pressure at high temperatures (≥∼1,000 °C), and cannot be produced by any conventional sintering techniques at the low pressures.

Precise measurements of sound velocities of high-pressure minerals are important to constrain the composition and constitution of the Earth's deep interior via comparisons with seismologically derived velocity models^28^. Transparent samples are needed for the measurements of sound velocities by Brillouin scattering technique^29^, and its application has mostly been limited to single crystals due to the unavailability of highly transparent polycrystalline bulk samples of high-pressure minerals suitable for such measurements, except for very thin samples^30^. Techniques for ultrasonic measurements in GHz regime have also been developed for sound velocity measurements of tiny samples^31^ such as synthesized at high pressure and temperature. Its application to polycrystalline samples, however, has not been made except for a recent study on NPD 32, as it requires well-sintered specimens with grain sizes <∼1 μm to avoid the effect of grain boundary scattering of the ultrasonic waves in the GHz range.

The ultrahigh-pressure conversion technique provides specimens ideal for these sound velocity measurements, and thereby greatly contributes to the mineralogical studies of the Earth's interior. Moreover, this technique provides super-transparent nano-crystalline ceramics with enhanced hardness, which are important for optical devices, including windows and lenses used in severe environments under high pressure, temperature and erosive conditions. Ease of doping minor elements in glass starting material for the ultrahigh-pressure conversion is also potentially important in making super-transparent ceramics for lasers, scintillators and other photonic devices, as well as for gem stones. Furthermore, applicability of the present technique to high-pressure phases with various crystal structures and advanced physical properties would lead to completely new and unique functional ceramics for scientific and industrial applications.

## Methods

### High-pressure experiments

We used bulk glass starting material with a composition of Ca_3_Al_2_Si_3_O_12_, which was made by de-carbonation and subsequent melting of a mixture of CaCO_3_, Al_2_O_3_ and SiO_2_ in a furnace, followed by quenching under air to suppress development of cracks. The bulk glass, without any visible cracks and bubbles, was cored with an ultrasonic machining device to make a rod with a diameter of 4.5 mm and a length of 3 mm. The glass rod starting material was wrapped with a thin foil (thickness of 50 μm) of pure iron, which reacts with a trace amount of water from air that could be present on the surface of the rod samples according to the reaction Fe+H_2_O→FeO+FeH_*x*_ under the present pressure and temperature conditions^33^. The sample was further enclosed in a gold capsule and inserted into the cell assembly shown in Supplementary Fig. 1, which was dried at 150 °C in an oven before being assembled. All the procedures of assembling Au capsule, MgO electric and LaCrO_3_ thermal insulators, Pt heater and MgO–CoO pressure medium were made on a hot plate at 150 °C. The cell assembly was kept in the oven until commencement of the ultrahigh-pressure synthesis runs.

The cell suffered pressures of 5–15 GPa and temperatures of 1,100–1,600 °C for 2 h in multianvil apparatus. Temperature was then decreased to 800 °C, and pressure was slowly released while the temperature was kept constant to reduce deviatoric stress in the sample so that development of cracks was minimized on decompression. The recovered samples at the ambient pressure were analysed using various techniques.

The ultrahigh-pressure synthesis of grossular was made with a Kawai-type 6–8 multianvil apparatus^34^ operated in a 3,000 ton press (ORANGE-3000) at the Geodynamics Research Center. To secure the larger sample volumes, we did not insert thermocouple wires to measure temperature in the high-pressure cell, and instead the temperature was evaluated by the predetermined electric power–temperature relations at 5, 10 and 15 GPa. Because of some difference in the temperature generation efficiency due to different deformation behaviours of Pt foil heater under pressure from run to run, temperature uncertainties of ∼5% of the nominal values are unavoidable. Pressure was estimated based on the phase transitions in some reference materials, and may also suffer uncertainties of ∼5%.

The bulk glass samples of garnet stoichiometry with other compositions, such as Mg_3_Al_2_Si_3_O_12_ (pyrope) and solid solutions between Ca_3_Al_2_Si_3_O_12_ and Ca_3_Cr_2_Si_3_O_12_ (uvarovite) and those between Mg_3_Al_2_Si_3_O_12_ and Mg_3_Cr_2_Si_3_O_12_ (knorringite) were also used as the starting materials for comparison. The bulk glass starting materials were prepared using the same procedures as those used in making the bulk glass with the grossular composition. For the synthesis of larger and crack-free samples of garnets with the compositions shown in Fig. 6, we used a multianvil apparatus operated in a 6,000 ton press (BOTCHAN-6000)^13^ at the Geodynamics Research Center. The design of the cell assembly adopted for these runs was virtually the same as shown in Supplementary Fig. 1 (except for the dimensions), but we used a softer NaCl, instead of MgO, as an electric insulator for the metal capsule to reduce the influence of deviatoric stress in the sample on the release of pressure. The grain sizes of the synthesized pyrope-rich garnets at 15 GPa and 1,400 °C were somewhat larger than those of grossular after the high-pressure and high-temperature synthesis for 2 h, but these may be reduced to <100 nm by adopting shorter run durations.

### Phase identification and chemical analysis

The recovered samples were first examined by an X-ray diffractometer (Rigaku, Rapid II) operated at 40 kV and 30 mA for phase identification. A Cu-Kα radiation (collimated to ∼100 μm) was used for the measurements and the diffraction patterns were recorded on an imaging plate. The 2*θ*-intensity profiles were obtained by integrating the pixel intensity over definite angular sectors. The samples below 1,200 °C remained glass with the characteristic halo background in the X-ray diffraction profiles, but those at and above 1,200 °C at all pressures were identified as pure garnet based on identification of all the diffraction peaks.

Field emission-SEM (scanning electron microscope; JEOL, JSM-7000F) with an energy-dispersive X-ray spectrometer (Oxford, X-Max 20) was used for micro-structure and chemical composition analyses. Before analyses, the mirror-polished samples were coated with carbon of ∼25 nm thick to prevent charging. Chemical quantification analysis was conducted at a beam current of 1 nA with an accelerating voltage of 20 kV, leading to the results that show the glass starting material and the synthesized grossular have identical bulk compositions of Ca_3_Al_2_Si_3_O_12_ within the uncertainties of the measurements (<0.5%).

### Microstructure observation

The grain boundaries were not clearly observed on the polished surface in most of the polycrystalline garnet samples using the field emission-SEM, except for the samples synthesized at 5 GPa and that at 1,200 °C, at 15 GPa. For grain size measurements of some samples composed of micrometre-size grains, fracture surfaces were prepared and coated with osmium of ∼5 nm thick to minimize charging effect.

Microtexture analysis of the samples was also performed by using a TEM (JEOL, JEM-2100 F) operated at 200 kV. Thin cross-section foils of about 10 × 5 μm with a thickness of 0.1–0.2 μm were prepared by using a focus-ion beam (JEOL, JEM-9310FIB) system. Any residual pores in the transparent samples were not detected by TEM observations, as shown by the high-resolution images of such samples (Fig. 2a,b). This is reasonable for the samples synthesized at such ultrahigh pressures and high temperatures in the present study as the pores can be totally collapsed in the sintered samples at pressures above ∼2 GPa (ref. 35), in addition to the use of bulk glass starting material without any visible cracks and bubbles. The porosities of some of the transparent samples determined by the Archimedes method were within the uncertainties of the measurements (∼0.5%) and are probably far less than these uncertainties and difficult to be evaluated by this method, as suggested in the case for NPD^32^. For the opaque sample obtained at 1,200 °C at 15 GPa (Fig. 1a), the presence of small patches of remnant glass was confirmed along some grain boundaries (Fig. 2c,d).

### Grain size distribution

Grain sizes of the polycrystalline samples composed mainly of grains of <200 nm were determined by direct measurement of 100 grains that appear black (that is, satisfying the Bragg condition) in the TEM images taken with an objective lens aperture. This procedure gave realistic size distributions of randomly referred grains (Fig. 1 and Supplementary Fig. 2). Grain sizes of the samples composed of larger (>200 nm) grains were measured by the intercept method for 100 grains using SEM (for grain sizes larger than 1,000 nm) and TEM images. The distributions of grain size for the samples synthesized at 1,200–1,600 °C at 15 GPa (Fig. 1) are shown in Supplementary Fig. 2 and Supplementary Table 1.

### Water content

Micro Fourier transform infrared (FT-IR; PerkinElmer, Spectrum One) spectroscopic observation was made on a polycrystalline garnet sample synthesized from a glass powder to evaluate its water content after polishing to a thin section, but no observable absorption bands relevant to the hydroxyl were detected, suggesting that the sample was almost completely dry within the detection limit (less than a few p.p.m.). This sample, synthesized at 15 GPa and at 1,400 °C, has an average grain size of ∼2 μm and should contain a larger amount of absorbed water as compared with the present samples synthesized from bulk glass starting material, indicating that quantitative evaluation of the water content cannot be made by the FT-IR measurement. As the bulk glass was obtained by quenching under air, instead of using the conventional procedure of dropping the sample into water, and very careful treatment in excluding absorbed water was made throughout the experiments, we believe virtually no water is present in the present polycrystalline garnet samples.

### Hardness

The hardness of the recovered samples was measured by a durometer with a Knoop indenter (Shimazu, HMV-G), which was pushed on the polished surface of the sample with a load of 0.98 N for 15 s. A standard metal block with hardness of 9.0 GPa (in Vickers scale) was used as the reference, yielding the Knoop hardness of 8.8±0.1 GPa in the present measurement, which is slightly lower but marginally consistent with the recommended value. Knoop hardness of a single crystal of natural grossular with small amounts of Fe (FeO=∼0.6 wt%) and Mn (MnO=∼0.4 wt%) was measured by the same method, resulting in the hardness of 10.7±0.3 GPa, which is also slightly lower than that measured in Vickers hardness of 10.9±0.4 GPa. The lower values of Knoop hardness relative to those of Vickers hardness are consistent with the general trend for hard ceramics^36^, and we used the Knoop hardness throughout the present measurements without any corrections based on the measurement on the standard metal block. The measurement on the synthesized grossular samples was repeated on 10–15 points for each sample, and the results were averaged and shown in Supplementary Table 1 with the s.d.'s. It should be noted that the hardness of a single crystal may vary depending on the crystal plane and direction^37^. Nevertheless, the hardness of the single-crystal grossular is consistent with those of the polycrystalline samples with grain sizes far larger than 1 μm within the uncertainties (Fig. 4).

### Optical transmittance

Transmittance of lights in an ultraviolet to visible range (200–800 nm) was measured using a spectrophotometer (JASCO, V-670) equipped with an integral sphere assembly to minimize the effect of light scattering on the mirror-polished grossular samples with a thickness of 1.0 mm. The data for 50 measurements were stacked to reduce the noise level. The roughness of the sample surface and its inclination to the incident light may affect the transmittance, which is estimated to be of the order of 2–3% in the present measurements according to the reproducibility of the measured transmittance.

### Data availability

No data sets were generated or analysed during the current study, but the original data in this article and some of the original samples are available from the corresponding author on reasonable request.

## Additional information

**How to cite this article**: Irifune, T. *et al*. Pressure-induced nano-crystallization of silicate garnets from glass. *Nat. Commun.* **7**, 13753 doi: 10.1038/ncomms13753 (2016).

**Publisher's note**: Springer Nature remains neutral with regard to jurisdictional claims in published maps and institutional affiliations.

## Supplementary Material

Supplementary Information Supplementary Figures 1 & 2 and Supplementary Table 1.

Peer Review File
