# Supplementary material for: Pressure-induced nano-crystallization of silicate garnets from glass
Source: Nat Commun. 2016 Dec 7;7:13753. doi: 10.1038/ncomms13753 (PMC5151095; doi:10.1038/ncomms13753)
Supplement: Supplementary Information — Supplementary Figures 1 & 2 and Supplementary Table 1. [file ncomms13753-s1.pdf]

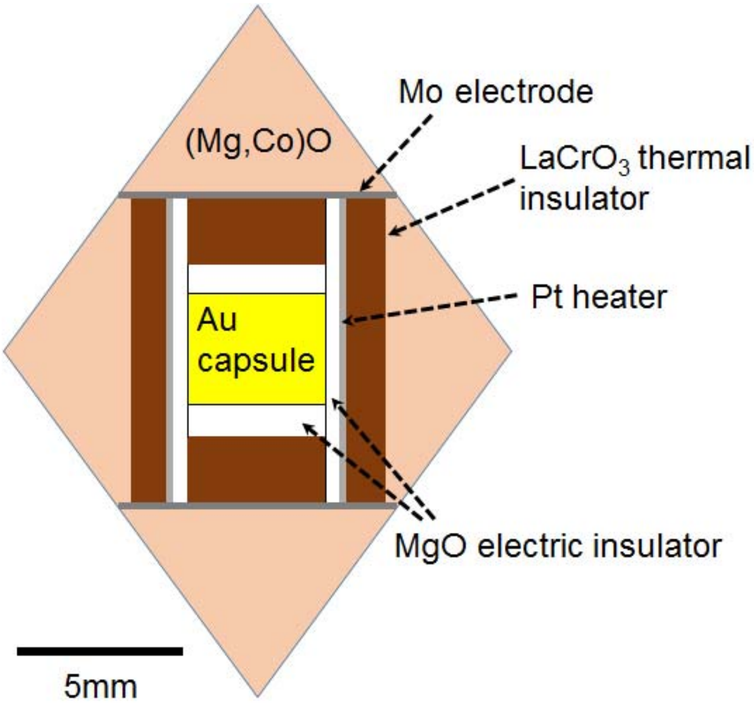

Supplementary Figure 1. A schematic illustration of a cross section of the furnace assembly used in the ultrahigh pressure synthesis. The starting glass rod, surrounded by a Fe foil, is enclosed in the Au capsule. Enlarged cell assembly was used for synthesis of other garnets, but the basic design of the assembly is identical to that shown here, except for MgO used as the electric insulator, which was replaced by softer NaCl.

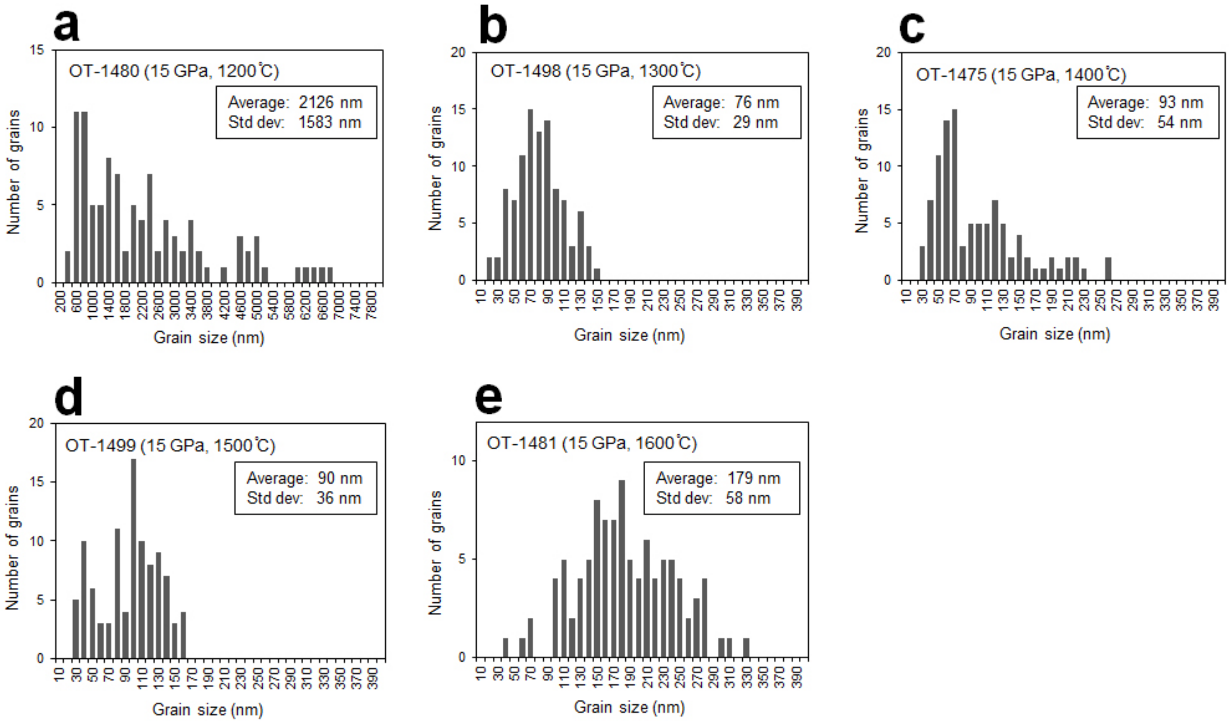

Supplementary Figure 2. Grain size distributions of the polycrystalline grossular samples synthesized at 15 GPa at 1200-1600ºC. The measurements on the sample synthesized at 1200ºC were made using mainly the SEM image, while those on other samples were based on TEM images. **a-e** correspond to the samples shown in Fig.1a-e.

Supplementary Table 1. Experimental conditions and results of the synthesis of grossular.

Run	Press. (GPa)	Temp. (ºC)	Grain Size (nm)	Knoop Hardness (GPa)
OT-1773	5	1100	GL	N.A.
OT-1526	5	1200	33142 (13236)	11.6 (0.6)
OT-1518	5	1400	5252 (1565)	11.5 (0.6)
OT-1529	5	1600	7341 (3241)	11.8 (0.7)
OT-1780	8	1200	341 (169)	11.0 (0.7)
OT-1793	8	1300	647 (289)	11.7 (0.1)
OT-1806	8	1400	889 (380)	11.9 (0.5)
OT-1662	10	1100	GL	6.5 (0.4)
OT-1581	10	1200	199 (41)	12.6 (1.0)
OT-1710	10	1300	149 (38)	13.3 (0.7)
OT-1582	10	1400	30 (6)	14.4 (0.5)
OT-1679	10	1500	156 (31)	N.A.
OT-1709	10	1600	172 (30)	N.A.
OT-1769	12	1400	50 (11)	14.5 (1.1)
OT-1562	15	1100	GL	6.6 (0.2)
OT-1480	15	1200	2126 (1583)	N.A.
OT-1498	15	1300	76 (29)	12.1 (0.8)
OT-1475	15	1400	93 (54)	14.6 (0.6)
OT-1499	15	1500	90 (36)	13.7 (0.7)
OT-1481	15	1600	179 (58)	12.4 (0.6)
