# Supplementary material for: Pressure-induced nano-crystallization of silicate garnets from glass
Source: Nat Commun. 2016 Dec 7;7:13753. doi: 10.1038/ncomms13753 (PMC5151095; doi:10.1038/ncomms13753)
Supplement: Peer Review File [file ncomms13753-s2.pdf]

Editorial Note: this manuscript has been previously reviewed at another journal that is not operating a transparent peer review scheme. This document only contains reviewer comments and rebuttal letters for versions considered at Nature Communications.
